# Idiopathic pulmonary fibrosis: Physician and patient perspectives on the pathway to care from symptom recognition to diagnosis and disease burden

**DOI:** 10.1111/resp.14154

**Published:** 2021-10-05

**Authors:** Lisa Lancaster, Francesco Bonella, Yoshikazu Inoue, Vincent Cottin, James Siddall, Mark Small, Jonathan Langley

**Affiliations:** ^1^ Division of Allergy, Pulmonary and Critical Care Medicine, Department of Medicine Vanderbilt University Medical Center Nashville Tennessee USA; ^2^ Center for Interstitial and Rare Lung Diseases, Pneumology Department, Ruhrlandklinik University Hospital University Duisburg‐Essen Essen Germany; ^3^ Clinical Research Center National Hospital Organization Kinki‐Chuo Chest Medical Center Osaka Japan; ^4^ National Coordinating Reference Center for Rare Pulmonary Diseases, Louis Pradel Hospital University of Lyon Lyon France; ^5^ Respiratory Research Adelphi Real World Bollington UK; ^6^ Development, Medical Affairs Galapagos NV Mechelen Belgium

**Keywords:** interstitial lung disease, pulmonary fibrosis, quality of life, rare lung diseases, respiratory lung function tests

## Abstract

**Background and objective:**

Idiopathic pulmonary fibrosis (IPF) is a chronic progressive disease that requires ongoing care and is associated with considerable socioeconomic burden. We evaluated the IPF care pathway from symptom recognition to treatment. We describe the impact of IPF on healthcare resource use (HCRU), quality of life (QoL) and work impairment, and report differences in patient and physician perspectives using real‐world data from France, Germany, Japan and the United States.

**Methods:**

Quantitative, point‐in‐time data were collected as part of the Adelphi IPF II Disease Specific Programme™. Physician‐reported data (patient demographics, medical history, diagnoses, treatment) were matched to patient‐reported data (HCRU, QoL, work impairment). HCRU was measured as physician visits and hospitalizations. QoL and work impairment were measured using the EuroQol‐5 Dimensions (EQ‐5D) and Work Productivity and Activity Impairment questionnaires.

**Results:**

Overall, 244 physicians reported data on 1249 patients, 739 of whom self‐reported data. Diagnostic delays of 0.8 (Germany) to 2.0 (Japan) years after symptom onset were reported; treatment initiation was further delayed. In all countries, patients more often reported symptoms in the survey than did their physicians. On average, patients underwent 7–10 clinical tests before diagnosis. Antifibrotic use increased from 57% (2016) to 69% (2019); only 50% of patients with moderate/severe IPF were satisfied with their treatment. The 12‐month hospitalization rates were 24% (Japan) to 64% (United States). Patients reported low QoL (mean EQ‐5D visual analogue scale: 61.7/100).

**Conclusion:**

Patients with IPF experience considerable diagnostic and treatment delays. More effective therapies and management are needed to reduce the disease burden.

## Graphical abstract

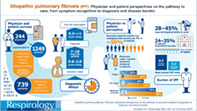

Patients with idiopathic pulmonary fibrosis experience diagnostic delays that can negatively influence their prognoses. This study uses real‐world data from four countries to characterize the considerable diagnostic and treatment delays based on patients' experience. We describe the resultant socioeconomic burden, including effects on healthcare resource utilization, quality of life and work impairment.

**See related** Editorial

## INTRODUCTION

Idiopathic pulmonary fibrosis (IPF) is a rare, progressive disease characterized by irreversible loss of lung function due to fibrosis, manifesting as increased coughing and dyspnoea, impairing quality of life (QoL).^1^, ^2^ Prognosis is poor (mean survival of 4 years) if untreated,^3^ and patients frequently experience diagnostic delays that can negatively affect prognosis.^4^, ^5^, ^6^ Patients rely primarily on antifibrotic therapy plus several supportive treatments but, despite recent advances, current therapies fail to halt disease progression and have limited impact on QoL.^2^ Reliance on healthcare services is considerable, contributing to a marked socioeconomic burden.^7^, ^8^ Patients with IPF require routine monitoring and multidisciplinary care,^9^, ^10^ and hospitalization is common, with many patients experiencing repeated admissions towards the end of life.^11^, ^12^

This study sought to characterize the real‐world pathway to care, from symptom recognition to diagnosis and treatment, for IPF in four countries; to describe the burden of IPF and its impact on healthcare resource use (HCRU), QoL and work productivity; and to report the perspectives of patients and physicians.

## METHODS

### Study design

This was a large, quantitative, point‐in‐time survey conducted between November 2018 and May 2019 among patients with IPF and their physicians in France, Germany, Japan and the United States, as part of the Adelphi IPF II Disease Specific Programme (DSP™; 2019 database).^13^ Patient record forms were prospectively completed online by pulmonologists for new or existing patients with IPF visiting for routine care. Physician recruitment in the DSP™ has been described^13^; briefly, physicians were identified from public lists of healthcare professionals (HCPs) and their patient loads were assessed. The patients were invited to participate in the DSP™ if they met the predefined eligibility criteria. The patient record form included questions on patient demographics, medical history, HCRU (number of HCP visits and hospitalizations due to IPF in the past 12 months), comorbidities, diagnosis, history of any IPF treatment, symptoms and severity based on both physician perception and lung function (most recent forced vital capacity [FVC in litres] results collected and converted to relative predicted percentages using appropriate reference values for each patient^14^). Predicted FVC definitions describing lung impairment were: mild >75%, moderate 50%–75% and severe <50% (hereafter referred to as mild, moderate and severe IPF, respectively).

Patients for whom physicians supplied data could voluntarily complete a self‐reported questionnaire (offline) collecting further information on medical history, diagnosis, symptoms and treatment satisfaction; these self‐reported patient data were matched against patient data supplied by physicians. As per Adelphi Real World standard operating procedures, the research was conducted as a survey in accordance with the amended Declaration of Helsinki, adhering to the International Chamber of Commerce/ESOMAR International Code on Market, Opinion and Social Research and Data Analytics,^15^ and the Health Insurance Portability and Accountability Act. The survey was submitted to the Western Institutional Review Board, a central international review board; an ethics exemption determination was granted on 29 November 2018 (Study Number: 1‐1135198‐1).

The patient questionnaire also included two validated patient‐reported outcome measures. QoL was assessed via the EuroQol‐5 Dimensions (EQ‐5D) questionnaire (three‐level, 3L; and visual analogue scale, VAS).^16^ The EQ‐5D‐VAS data for IPF were compared with data from the Adelphi non‐small cell lung carcinoma (NSCLC; 2016, Germany only), chronic obstructive pulmonary disease (COPD; 2018) and asthma (2018) DSP™ databases. The Work Productivity and Activity Impairment (WPAI) questionnaire was used to record the number of hours of work missed due to IPF, and the extent to which IPF affected productivity as a percentage impairment score. QoL, work impairment and HCRU data were used to characterize the socioeconomic burden of IPF.

### Statistical analysis

Descriptive analyses were generated using IBM SPSS Data Collection Survey Reporter between April 2019 and July 2019.

## RESULTS

### Study population

Of the 5193 physicians approached, 244 (4.7%) reported data on 1249 patients with IPF (Table 1). Of these patients, 739 self‐reported data. Patients were predominantly male, with a mean (SD) age at consultation of 67.8 (10.1) years. While few patients were current smokers at the time of the survey, 40% (United States) to 68% (Japan) previously smoked.

**TABLE 1:** Patient demographics and characteristics^a^ (physician‐reported data except where indicated otherwise)

Characteristics as reported by physicians	France (*n* = 301)	Germany (*n* = 300)	Japan (*n* = 256)	United States (*n* = 392)	Total (*N* = 1249)
Male sex, %	73	64	77	62	68
Mean (SD) age at symptom onset, years^b^	60.2 (13.2)	60.1 (8.3)	67.0 (10.8)	63.6 (9.9)	62.6 (10.7)
Mean (SD) age at consultation, years	67.3 (12.5)	65.0 (7.8)	73.4 (12.1)	66.8 (8.5)	67.8 (10.1)
Mean (SD) age at diagnosis, years	65.9 (11.50)	62.2 (9.16)	70.2 (9.33)	65.0 (10.65)	65.6 (10.61)
Smoking history, %					
Current smoker	9	7	2	4	5
Previous smoker	50	46	68	40	47
Never smoked	41	47	30	55	43
Mean (SD) length of time since patient stopped smoking, years	12 (10)	10 (9)	14 (11)	15 (13)	13 (11)
Employment status, %					
Unemployed	4	2	8	7	5
Homemaker	3	5	15	8	8
Long‐term sick leave	7	6	1	2	4
Part‐time employment	4	14	5	14	10
Full‐time employment	12	20	14	22	18
Retired	69	53	56	47	56
Caregiver required, %	17	16	14	24	18
Comorbidities, %					
Lung cancer	1	0	6	0	2
Pulmonary hypertension	3	5	0	2	3
Hypertension	29	49	27	32	34
GORD	20	11	5	18	14
Hypercholesterolaemia/hyperlipidaemia	9	17	5	11	11
Diabetes	8	18	9	11	12
Coronary artery disease	6	17	3	10	10
Chronic pulmonary disease	5	10	7	12	9
Arthritis	0	6	<1	19	7
Anxiety	11	2	1	10	6
Cardiac arrhythmia	7	8	4	5	6
Depression	6	5	2	9	6
None	27	17	36	14	22

*Note*: A total of 739 patients self‐reported data (France: *n* = 123; Germany: *n* = 233; Japan: *n* = 198; United States: *n* = 185).

Abbreviation: GORD, gastro‐oesophageal reflux disease.

^a^ Patient characteristics were recorded at the time of the survey; percentages may not sum to 100 due to rounding.

^b^ Patient‐ and physician‐reported data.

### Diagnostic journey

**FIGURE 1:**
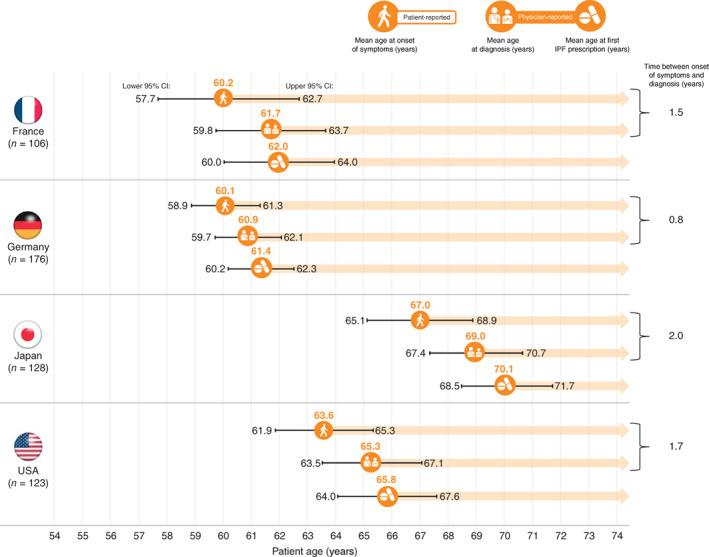
Key time points in the IPF patient journey (patient‐ and physician‐reported data). IPF, idiopathic pulmonary fibrosis

The mean age at symptom onset was lower in France and Germany than in Japan and the United States. There were considerable delays in time from symptom onset to diagnosis, varying from 0.8 years (Germany) to 2.0 years (Japan) (Figure 1). Across all patients, the median (range) time from symptom onset to seeking medical care was 6 (0–60) months, and 7 (0–90) months for time from the first physician visit to diagnosis. The mean time from diagnosis to the first IPF prescription ranged from ~0.3 years (France) to >1 year (Japan).

In patient record forms, physicians reported shortness of breath (SOB) during exertion before/at IPF diagnosis in 75% of patients, with dry cough in 50% and resting SOB in 27% (Figure S1 in the Supporting Information). Up to 45% of patients with early‐stage IPF may have been misclassified as having COPD because of the presence of similar generic respiratory symptoms. Other conditions suspected or investigated before a confirmed IPF diagnosis included congestive heart failure, gastro‐oesophageal reflux disease, asthma and acute bronchitis.

On average, diagnosis involved 7–10 different clinical tests per patient, most commonly HRCT (80%) and pulmonary function tests (73%); lung biopsy was less frequent (20%) (Table S1 in the Supporting Information). HRCT and chest radiographs were common across all countries. Spiral computed tomography (CT) was more common in France, and arterial blood gas and bronchoscopy were more common in France and Germany. Primary care physicians were usually the first HCP to see symptomatic patients about their breathing conditions before IPF diagnosis, with pulmonologists mainly responsible for diagnosis and treatment.

### Clinical burden

**FIGURE 2:**
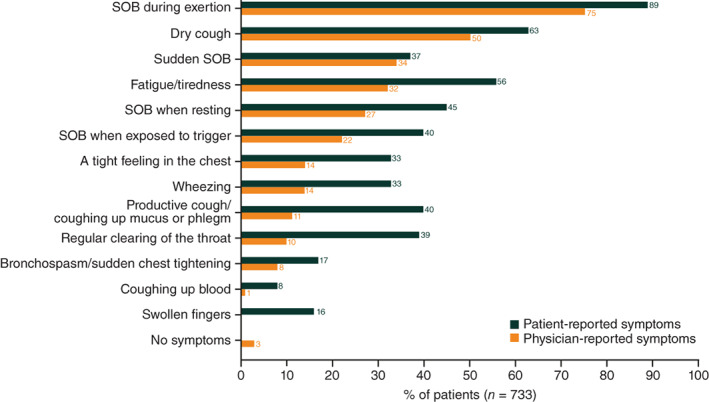
Most common symptoms associated with idiopathic pulmonary fibrosis (physician‐ and patient‐reported data). SOB, shortness of breath

Physicians generally under‐reported symptoms compared to patients (Figure 2), a trend observed across all countries (Figure S2 in the Supporting Information). The symptom most frequently reported by both physicians and patients was SOB during exertion (75% vs. 89%), followed by dry cough (50% vs. 63%). Fatigue was similarly under‐reported by physicians.

**FIGURE 3:**
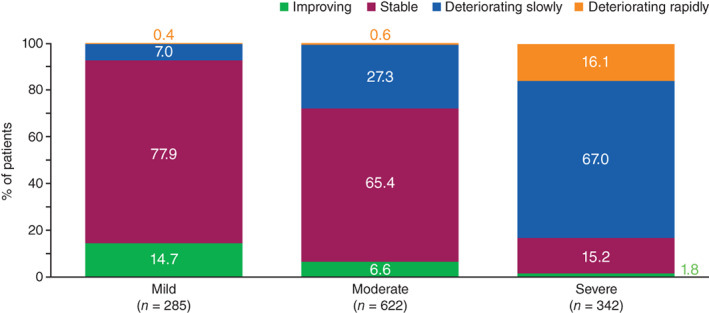
Physician‐perceived rate of idiopathic pulmonary fibrosis deterioration according to physician‐perceived disease severity

Physician‐reported IPF severity was misaligned with that determined by FVC in ~50% of patients. Across the countries assessed, 24%–34% of patients were considered by their physicians to have severe IPF, whereas 12%–44% of patients had a FVC <50%. Patients with physician‐perceived mild or moderate IPF were more likely to be classified as stable/improving than those with severe disease as indicated by FVC (Figure 3).

### Treatment

Antifibrotic use increased from 57% in 2016 to 69% in 2019; this trend was apparent in all countries and across all physician‐perceived disease severities, except for patients with mild IPF in France (Table 2). The largest increase in antifibrotic use was seen in patients with mild IPF in the United States (45% in 2016 to 81% in 2019). Only 47%–61% of patients with moderate IPF and 13%–41% with severe IPF based on FVC data were satisfied with their current treatment (Figure S3 in the Supporting Information).

**TABLE 2:** Percentage of patients receiving antifibrotic treatment (2016 vs. 2019) according to physician‐perceived severity of idiopathic pulmonary fibrosis, by country

Physician‐perceived severity	France (*n* = 301)	Germany (*n* = 300)	Japan (*n* = 256)	USA (*n* = 392)	Total (*N* = 1249)
Mild					
2016	57	34	25	45	38
2019	52	59	37	81	56
Moderate					
2016	69	65	52	60	62
2019	77	71	65	67	70
Severe^a^					
2016	56	78	79	62	67
2019	78	84	82	66	76
All					
2016	63	62	47	57	57
2019	72	72	60	69	69

*Note*: Pirfenidone and nintedanib were available in all four countries in both 2016 and 2019. In Japan and the United States, both treatments were available to all patients with idiopathic pulmonary fibrosis. Both treatments in France, and pirfenidone in Germany, were restricted to patients with a forced vital capacity ≥50% and diffusing capacity for carbon monoxide ≥30% of predicted value.

^a^ Physician‐perceived severe and very severe patients were grouped as ‘severe’.

### Burden of IPF: HCRU, QoL and work impairment

**FIGURE 4:**
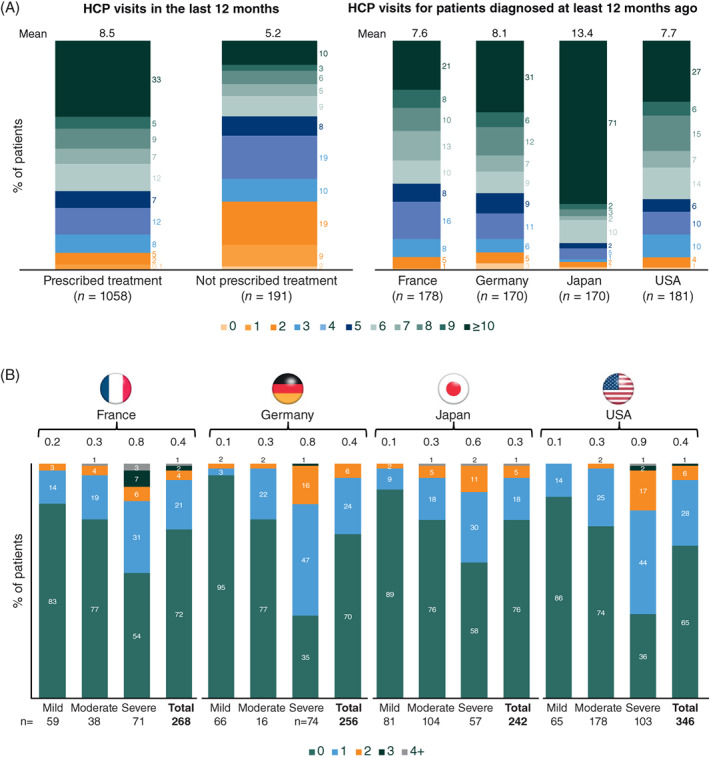
(A) HCP visits and (B) hospitalizations due to idiopathic pulmonary fibrosis in the last 12 months (physician‐reported data). Percentages have been rounded and may not sum to 100%. The number of hospitalizations shown may be an underestimation as the patients who died after an acute exacerbation/hospitalization were not captured in this survey. Disease severity was as stated by the physician for each patient. HCP, healthcare professional

Patients who were prescribed drug therapy (84.7%) consulted an HCP more frequently than untreated patients (8.5 vs. 5.2 visits/year). Within 12 months, 33% of treated patients and 10% of untreated patients underwent ≥10 visits; patients in Japan underwent the most physician visits (Figure 4A). Patients generally visited physicians for routine check‐ups (50%–67%) and repeat prescriptions (17%–43%) and rarely to discuss treatment issues (2%–11%). Overall, the 12‐month hospitalization rates ranged from 24% (Japan) to 35% (United States) (Figure 4B) and the mean duration of hospitalization was 8 nights. Hospitalization was more frequent when disease was perceived as severe by physicians, but still common (5%–17%) in patients with mild functional impairment (Figure 4B).

In all countries, patients with more severe IPF found their condition to be a major problem in daily life, negatively impacting their social life, relatives and friends (Table S2 and Figure S3 in the Supporting Information). Patients with IPF reported a disease burden similar to that of NSCLC and worse than moderate‐to‐severe COPD (Figure S4 in the Supporting Information). Mean EQ‐5D‐VAS scores decreased with increasing disease severity; scores for patients with moderate IPF were comparable to those for moderate COPD but worse than for moderate asthma, based on comparative real‐world data. The EQ‐5D‐3L values for patients with moderate IPF were worse than those for patients with moderate COPD and moderate asthma. Mean EQ‐5D‐3L was 0.71 in treated patients and 0.77 in untreated patients. Non‐retired patients with IPF (representing just under half of the study population) reported that their ability to work was impaired by 28%, 45%, 40% and 34% in France, Germany, Japan and the United States, respectively.

## DISCUSSION

We studied the real‐world pathway to care in IPF and assessed the disease burden from patient and physician perspectives. Despite the introduction of international guidelines in 2018 that established IPF diagnostic criteria,^17^ significant delays in time to diagnosis and time to treatment initiation persist, varying from 0.8 years in Germany to 2.0 years in Japan. Diagnostic delays of 1.5 to >2 years have been reported in Europe^18^, ^19^, ^20^ and of >1 year in the United States,^4^ and have been attributed to patients not meeting IPF diagnostic criteria early in the disease course, physicians dismissing symptoms and comorbidities.^4^, ^18^, ^20^ Differences in healthcare systems and patient cost burden between countries may impact the likelihood of patients seeking treatment and how promptly they receive it.^21^ Notably, diagnostic delay was greatest in Japan despite wide use of annual health check‐ups and CT. We suspect that, in Japan, asymptomatic early‐stage IPF may be detected during annual health check‐ups and confirmed by CT, but findings may be underestimated as interstitial pneumonia or overestimated as (unclassifiable) idiopathic interstitial pneumonia, then later confirmed as IPF due to progressive disease behaviour or at initiation of government‐funded antifibrotic treatment.

Misdiagnosis and delayed diagnosis were common in our study. Up to 45% of patients with early‐stage IPF were first misdiagnosed, in line with 41% reported previously.^18^ There are multiple possible explanations for this finding. The symptoms of IPF are similar to COPD and asthma, making diagnosis difficult.^22^, ^23^, ^24^ The most common symptoms (SOB, dry cough, fatigue) may be attributed to old age, smoking or common respiratory/cardiovascular diseases.

Unfamiliarity with IPF among general practitioners can delay diagnosis^25^ but can still occur even after imaging, lung function tests and pulmonologist evaluation. On average, patients in this survey underwent seven to 10 different diagnostic procedures before IPF was confirmed—more than the three to four suggested to be necessary by relevant guidelines.^12^, ^17^ Despite frequent use of HRCT and lung function tests, similar to diagnostic data reported in American and European registries,^4^, ^18^, ^19^, ^26^ diagnostic delays persisted. Analysis of US Medicare 2012 claims data showed that nearly one‐third of patients with IPF had their first CT scan >3 years before diagnosis, and 35% had seen a pulmonologist >3 years before diagnosis.^5^ Increased education and awareness of IPF by primary care physicians and improved testing are needed to shorten the time to diagnosis.

Disease progression and symptoms of IPF were underestimated by physicians, which may affect treatment. Physician‐reported severity was often misaligned with severity as determined by FVC, suggesting that physicians may be inadequately assessing symptoms and/or patients are poorly communicating their symptoms, but also that physicians may be unfamiliar with IPF and how symptoms correlate to FVC. Physicians may be using different measures (e.g., oxygen needs) to characterize IPF severity. Despite this, antifibrotic use increased from 2016 to 2019 irrespective of IPF severity. This upward trend in treatment was seen across all countries, although disparities exist: 81% of patients with mild IPF in the United States were receiving antifibrotic treatment at the time of survey versus 37% in Japan. These disparities can only be partially explained by country differences in antifibrotic indication and treatment; in the United States and Japan, antifibrotics are available to all patients with IPF, whereas in France and Germany, some antifibrotics are available only to patients with an FVC ≥50% and diffusing capacity for carbon monoxide ≥30% of predicted value. Low antifibrotic use in Japan may be associated with high rates of gastrointestinal side effects in this population^27^ and delays in patient financial support. Although most pulmonologists considered initiating antifibrotic therapy immediately after IPF diagnosis (81.7% in a recent US study),^28^ the proportion of patients receiving therapy is often substantially smaller.^29^ Pulmonologists who saw fewer patients with IPF were less comfortable discussing a patient's prognosis, believed less in the effectiveness of antifibrotic therapy, tended to adopt a ‘watch and wait approach’ and were more likely to wait >4 months between diagnosis and treatment initiation.^30^ Despite relatively high rates of antifibrotic treatment in the present study, approximately half of patients with moderate‐to‐severe IPF remained dissatisfied with their treatment; this may have been due to side effects of antifibrotics and/or the complexity of IPF care.^31^

IPF was associated with a marked socioeconomic burden, as reflected by the detrimental effects on QoL, healthcare utilization and the ability to work. The EQ‐5D‐VAS value of 61.7 observed here is similar to that reported in the German INSIGHTS‐IPF registry (mean 62.6, SD 18.5),^32^ but lower than that in the US IPF‐PRO registry (median 75.0, range 60.0–85.0).^33^ Evidence suggests that a difference as small as 0.5–5.0 units on the EQ‐5D‐VAS scale is clinically important in IPF.^34^ In this study, QoL was lower in patients with more severe IPF, aligning with registry data^32^, ^33^ and findings that QoL is particularly low in the last 2 years of life in patients with IPF.^35^ Furthermore, caregiver burden related to IPF is considerable and qualitative evidence suggests a need for improved caregiver support.^36^

In this study, patients receiving IPF treatment and those with more severe IPF relied more frequently on healthcare services. High HCRU in IPF, including recurrent hospitalizations, represents a substantial economic burden^8^, ^37^, ^38^, ^39^, ^40^; it accelerates beginning >1 year before diagnosis and remains elevated over the following 5 years.^41^ Per capita, the annual cost of IPF in the United States has been estimated to be 2.5–3.5 times higher than the national healthcare expenditure, and twice that estimated for COPD.^7^, ^42^ Moreover, patients with IPF reported higher impairment rates (28%–45%) than those reported for COPD (11%–28%) and sarcoidosis (13%–28%), further contributing to the economic burden of IPF.^42^, ^43^ WPAI data in other diseases estimate a minimal clinically important difference for work productivity loss in the range of 15%–20%.^44^, ^45^

Survey studies have some limitations. Patient characteristics were recorded at the time of survey (not at diagnosis) and therefore may have been affected by a patient's disease. Work impairment reports were limited to employed patients at the time of the survey; therefore, work impairment in patients who previously stopped working because of their IPF was not captured. Additionally, FVC was the only clinical marker of severity examined, although it is likely to be the most relevant comparison for physician‐perceived severity. Due to a low physician response rate in this study, included patients may not be representative of the general IPF population. Lastly, poor recall of symptom onset can introduce bias.

In conclusion, there is substantial need to optimize the current management approach for IPF given the rapidly progressive and irreversible nature of IPF, the considerable diagnostic delays that persist and the resulting disease burden. Efforts are required to reduce diagnostic uncertainty to ensure that patients receive earlier intervention, and to improve treatment satisfaction in terms of impact on symptoms, safety and emotional well‐being and QoL.

## CONFLICT OF INTEREST

This study was also previously presented at the 2020 Annual Congress of the European Respiratory Society. The research was funded by Galapagos NV (Mechelen, Belgium). The study sponsor, Galapagos NV (Mechelen, Belgium), played a role in the study design, data collection and analysis; decision to publish; and preparation of the manuscript. Medical writing support, including development of a draft outline and subsequent drafts in consultation with the authors, collating author comments, copyediting, fact checking and referencing, was provided by Kristian Clausen, MPH at Aspire Scientific Limited (Bollington, UK). Funding for medical writing support for this article was provided by Galapagos NV (Mechelen, Belgium).

Lisa Lancaster has received grant/research support from Bellerophon, Biogen, Boehringer Ingelheim, Celgene, Galactic, Galapagos NV, Genentech, Novartis, BMS, Fibrogen and Pliant; has worked as a paid consultant for AstraZeneca, DevProBiopharma, Galapagos NV, Genentech, United Therapeutics and Veracyte; and has been paid as a speaker (disease state education) by Boehringer Ingelheim, Genentech, United Therapeutics and Veracyte. Francesco Bonella has received grant/research support from Boehringer Ingelheim and Savara; has worked as a paid consultant for Boehringer Ingelheim, Bristol Myers Squibb, Galapagos, Roche and Savara; and has been paid as a speaker by Boehringer Ingelheim and Roche. Yoshikazu Inoue has worked as a paid consultant for Asahi Kasei, Boehringer Ingelheim, Galapagos, NITTO, Promedior, Roche, Shionogi and Taiho, and unpaid advisor for Savara; and has been paid as a speaker by Boehringer Ingelheim and Shionogi. Vincent Cottin has received grant/research support from Boehringer Ingelheim; has worked as a paid consultant for Roche/Promedior; has participated in advisory boards for Actelion, Bayer/MSD, Galapagos and Novartis; has received payment for travel to medical meetings from Actelion, Boehringer Ingelheim and Roche; has been paid as a speaker by Actelion, Boehringer Ingelheim, Novartis, Roche and Sanofi; and has served as a member/chair of data safety monitoring boards for Bayer/MSD, Celgene, Galapagos, Galecto and Promedior. Jonathan Langley is an employee and warrant holder at Galapagos NV. Mark Small and James Siddall are employees of Adelphi Real World, a company that received research funding from Galapagos NV for the current study.

## AUTHOR CONTRIBUTION

**Lisa Lancaster:** Conceptualization; data curation; formal analysis; funding acquisition; investigation; methodology; project administration; resources; software; supervision; validation; visualization; writing – original draft; writing – review and editing. **Francesco Bonella:** Conceptualization; data curation; formal analysis; funding acquisition; investigation; methodology; project administration; resources; software; supervision; validation; visualization; writing – original draft; writing – review and editing. **Yoshikazu Inoue:** Conceptualization; data curation; formal analysis; funding acquisition; investigation; methodology; project administration; resources; software; supervision; validation; visualization; writing – original draft; writing – review and editing. **Vincent Cottin:** Conceptualization; data curation; formal analysis; funding acquisition; investigation; methodology; project administration; resources; software; supervision; validation; visualization; writing – original draft; writing – review and editing. **James Siddall:** Conceptualization; data curation; formal analysis; funding acquisition; investigation; methodology; project administration; resources; software; supervision; validation; visualization; writing – review and editing. **Mark Small:** Conceptualization; data curation; formal analysis; funding acquisition; investigation; methodology; project administration; resources; software; supervision; validation; visualization; writing – original draft; writing – review and editing. **Jonathan Langley:** Conceptualization; data curation; formal analysis; funding acquisition; investigation; methodology; project administration; resources; software; supervision; validation; visualization; writing – original draft; writing – review and editing.

## HUMAN ETHICS APPROVAL DECLARATION

The research was conducted as a survey in accordance with the amended Declaration of Helsinki, adhering to the International Chamber of Commerce/ESOMAR International Code on Market, Opinion and Social Research and Data Analytics,^15^ and the Health Insurance Portability and Accountability Act. The survey was submitted to the Western Institutional Review Board, a central international review board; an ethics exemption determination was granted on 29 November 2018 (Study Number: 1‐1135198‐1). Informed consent for participation in the study was obtained from all patients.

## Supporting information

Supporting information.

**Visual Abstract** Idiopathic pulmonary fibrosis (IPF): Physician and patient perspectives on the pathway to care, from symptom recognition to diagnosis and disease burden
