# Supplementary material for: Idiopathic pulmonary fibrosis: Physician and patient perspectives on the pathway to care from symptom recognition to diagnosis and disease burden
Source: Respirology. 2021 Oct 5;27(1):66–75. doi: 10.1111/resp.14154 (PMC9135122; doi:10.1111/resp.14154)
Supplement: Supplementary file 1 — Supporting information. [file RESP-27-66-s001.doc]

**SUPPORTING INFORMATION**

# Idiopathic pulmonary fibrosis: physician and patient perspectives on the pathway to care, from symptom recognition to diagnosis and disease burden

Lisa LANCASTER,^1^ Francesco BONELLA,^2^ Yoshikazu INOUE,^3^ Vincent COTTIN,^4^ James SIDDALL,^5^ Mark SMALL^5^ and Jonathan LANGLEY^6^

**Authors’ affiliation(s):** ^1^Division of Allergy, Pulmonary and Critical Care Medicine, Department of Medicine, Vanderbilt University Medical Center, Nashville, TN, USA; ^2^Center for Interstitial and Rare Lung Diseases, Pneumology Department, Ruhrlandklinik University Hospital, University Duisburg-Essen, Essen, Germany; ^3^National Hospital Organization Kinki-Chuo Chest Medical Center, Osaka, Japan; ^4^National Coordinating Reference Center for Rare Pulmonary Diseases, Louis Pradel Hospital, University of Lyon, member of OrphaLung, RespiFil, ERN-LUNG, Lyon, France; ^5^Respiratory Research, Adelphi Real World, Bollington, UK; ^6^Development, Medical Affairs, Galapagos NV, Mechelen, Belgium

**Figure S1** Pre-diagnostic symptoms and conditions suspected before IPF confirmation, by country.


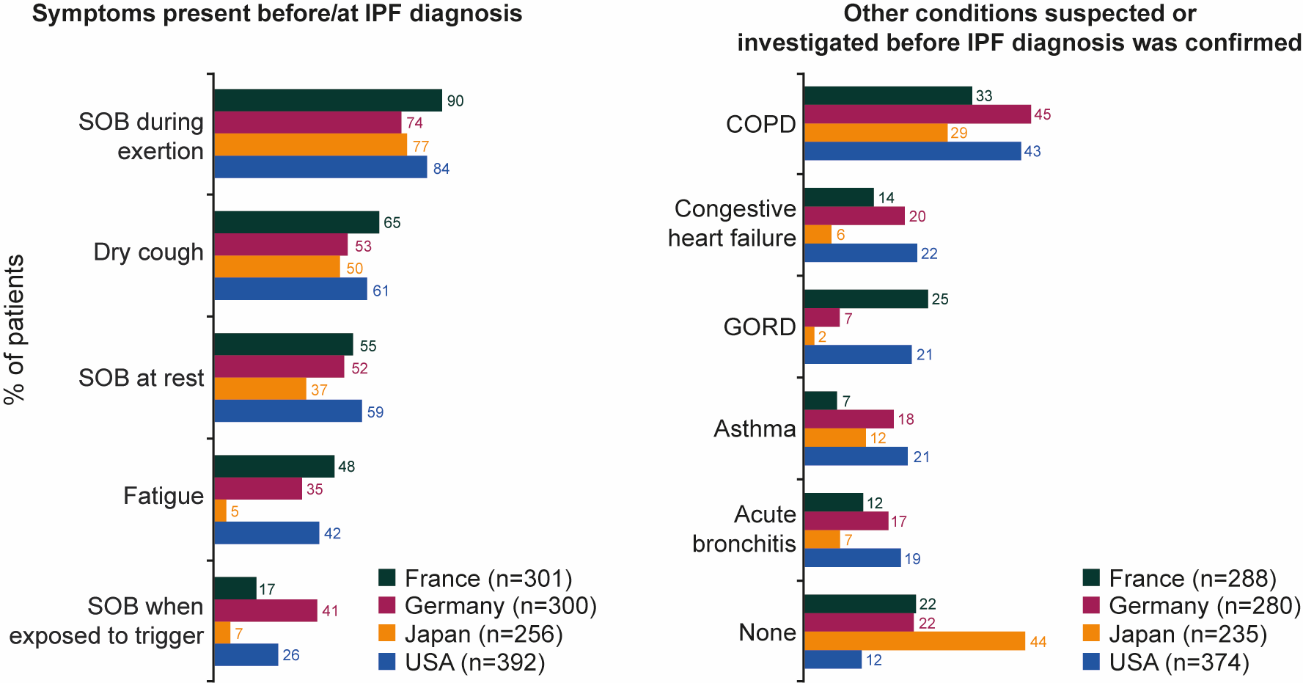


Physician-reported patient data.

COPD, chronic obstructive pulmonary disease; GORD, gastro-oesophageal reflux disease; IPF, idiopathic pulmonary fibrosis; SOB, shortness of breath.

**Figure S2** Most common symptoms associated with idiopathic pulmonary fibrosis by country.


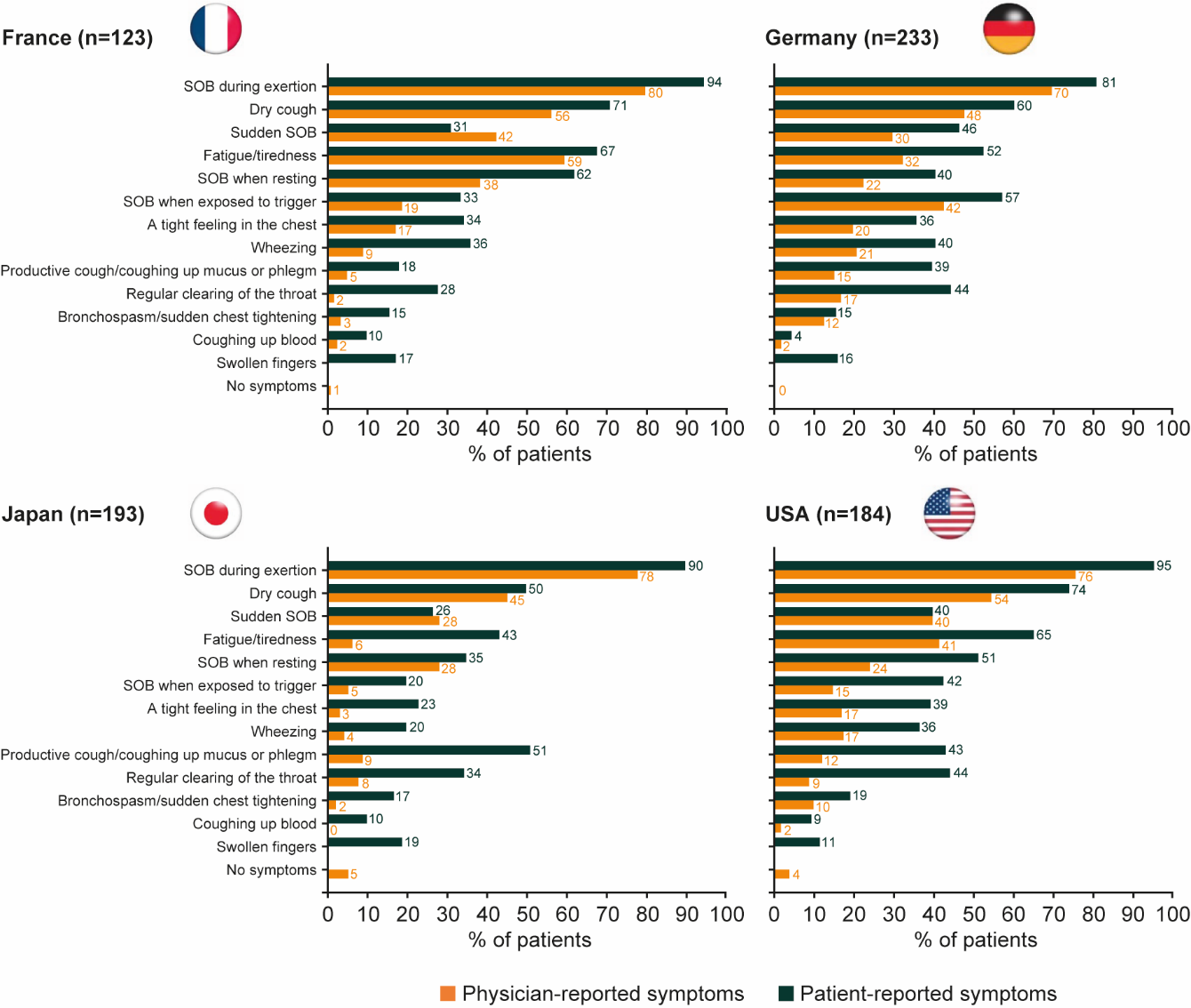


No patient data were available for ‘no symptoms’ and no physician data were available for ‘swollen fingers’; data for ‘coughing up blood’ were not available for Japan.

SOB, shortness of breath.

**Figure S3** Patient perception of IPF treatment and disease impact by disease severity (patient-reported data).


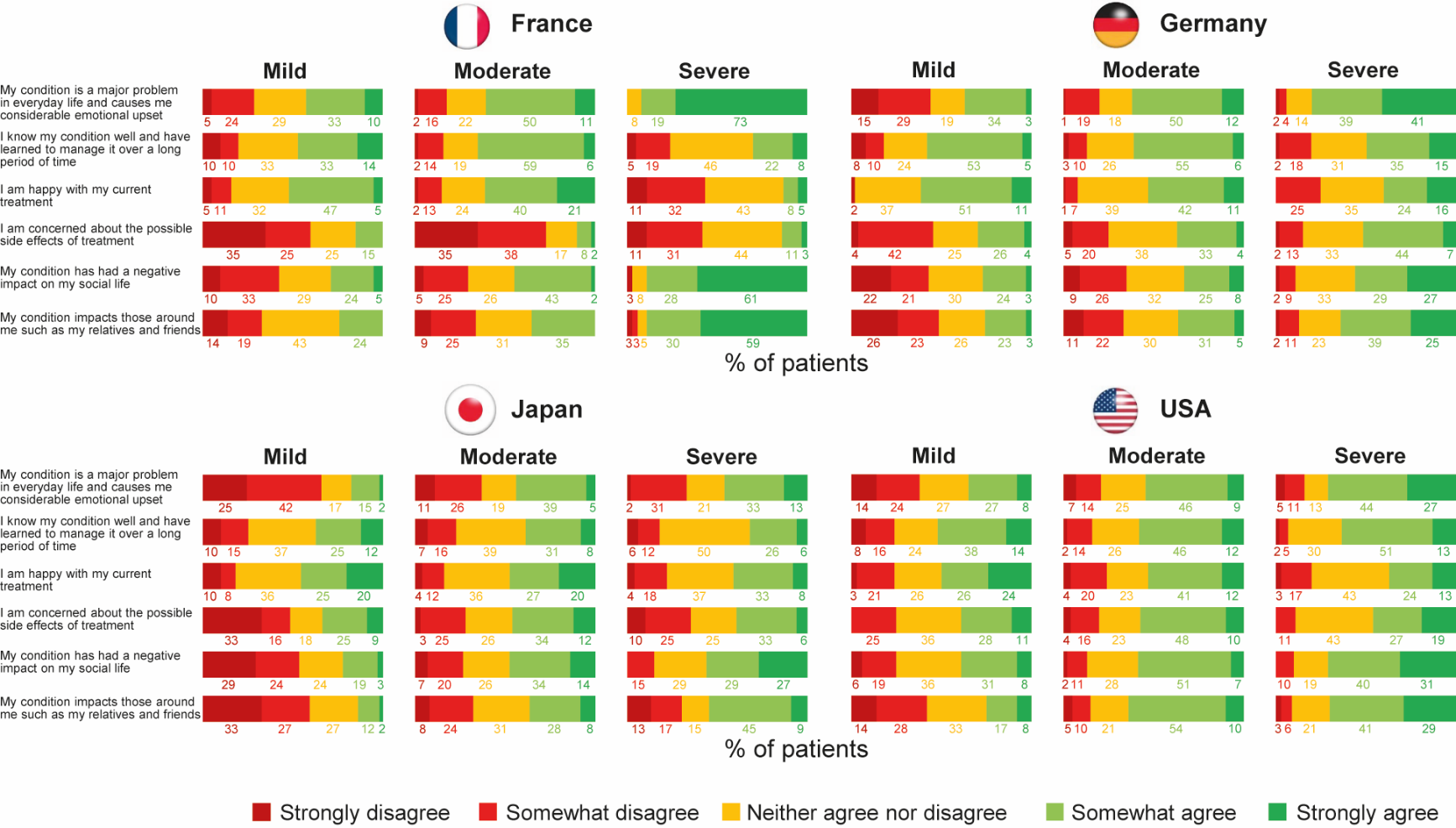


The figure illustrates disparities among countries in patient perceptions of IPF treatment and impact by disease severity. Generally, patients with more severe disease report a greater negative impact on their lives and are less likely to be satisfied with their current treatment.

IPF, idiopathic pulmonary fibrosis.

**Figure S4** Patient-reported EQ-5D-VAS scoring across the Adelphi Respiratory Disease Specific Programme™. Adelphi Real World Disease Specific Programmes™ [data on file] cited with permission.


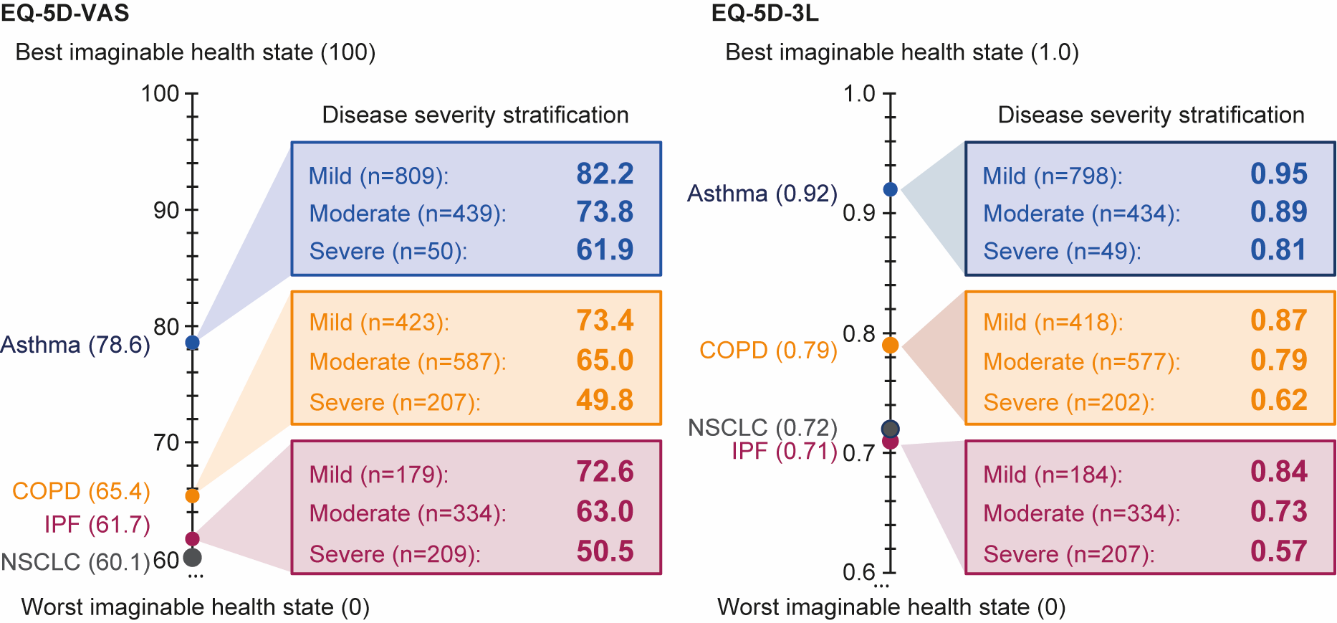


NSCLC data are from the 2016 Germany dataset only (EQ-5D-VAS, n=266; EQ-5D-3L, n=265), asthma and COPD data are from the 2018 datasets, and IPF data are from the 2019 dataset. Disease severity for IPF, asthma and COPD was as stated by the physician for each patient. Physicians' perception of severity was not reported for NSCLC.

3L, three-level descriptive system; COPD, chronic obstructive pulmonary disease; EQ-5D, EuroQol-5 Dimensions; IPF, idiopathic pulmonary disease; NSCLC, non-small cell lung cancer; VAS, visual analogue scale.

**Table S1** Diagnostic tests and responsibility for diagnosis and treatment decisions (physician-reported data).

| **Characteristic** | **France (n=301)** | **Germany (n=300)** | **Japan  (n=256)** | **USA  (n=392)** | **Total (n=1249)** |
| --- | --- | --- | --- | --- | --- |
| Diagnostic tests, % |  |  |  |  |  |
| HRCT | 83 | 77 | 84 | 77 | 80 |
| FEV | 80 | 80 | 66 | 68 | 73 |
| FVC | 88 | 67 | 69 | 69 | 73 |
| Chest radiograph | 66 | 60 | 79 | 63 | 66 |
| CO diffusing capacity | 87 | 56 | 53 | 64 | 66 |
| Arterial blood gas | 71 | 71 | 52 | 36 | 56 |
| Pulse oximetry | 37 | 35 | 62 | 54 | 47 |
| Spiral computed tomography | 74 | 37 | 27 | 39 | 45 |
| Bronchoscopy | 58 | 58 | 26 | 32 | 43 |
| 6-minute walk test | 57 | 42 | 35 | 38 | 43 |
| Lung biopsy | 22 | 23 | 13 | 21 | 20 |
| Mean (SD) number of diagnostic tests per patient | 9.5 (4.5) | 8.1 (3.2) | 6.6 (2.9) | 7.3 (4.6) | 7.9 (3.6) |
| PCP/pulmonologist, % |  |  |  |  |  |
| Seen prior to diagnosis | 76/55 | 64/43 | 34/70 | 65/72 | 61/59 |
| Responsible for diagnosis | 0/97 | 3/92 | 3/96 | 2/93 | 2/94 |
| Responsible for treatment decisions | 5/100 | 11/98 | 5/96 | 11/98 | 8/98 |

CO, carbon monoxide; FEV, forced expiratory volume; FVC, forced vital capacity; HRCT, high-resolution computed tomography; PCP, primary care physician; SD, standard deviation.

**Table S2** Mean EQ-5D-3L and EQ-5D-VAS scores by country (patient-reported data).

|  | **EQ-5D-VAS** | **EQ-5D-3L** |
| --- | --- | --- |
| France (n=120) | 57.6 | 0.518 |
| Germany (n=231) | 61.3 | 0.780 |
| Japan (n=194) | 65.0 | 0.739 |
| USA (n=180) | 61.5 | 0.727 |

3L, three-level descriptive system; EQ-5D, EuroQol-5 Dimensions; VAS, visual analogue scale.
