# Supplementary material for: Idiopathic pulmonary fibrosis: Physician and patient perspectives on the pathway to care from symptom recognition to diagnosis and disease burden
Source: Respirology. 2021 Oct 5;27(1):66–75. doi: 10.1111/resp.14154 (PMC9135122; doi:10.1111/resp.14154)
Supplement: Supplementary file 2 — Visual Abstract Idiopathic pulmonary fibrosis (IPF): Physician and patient perspectives on the pathway to care, from symptom recognition to diagnosis and disease burden [file RESP-27-66-s002.pdf]

Idiopathic pulmonary fibrosis (IPF): Physician and patient perspectives on the pathway to care, from symptom recognition to diagnosis and disease burden

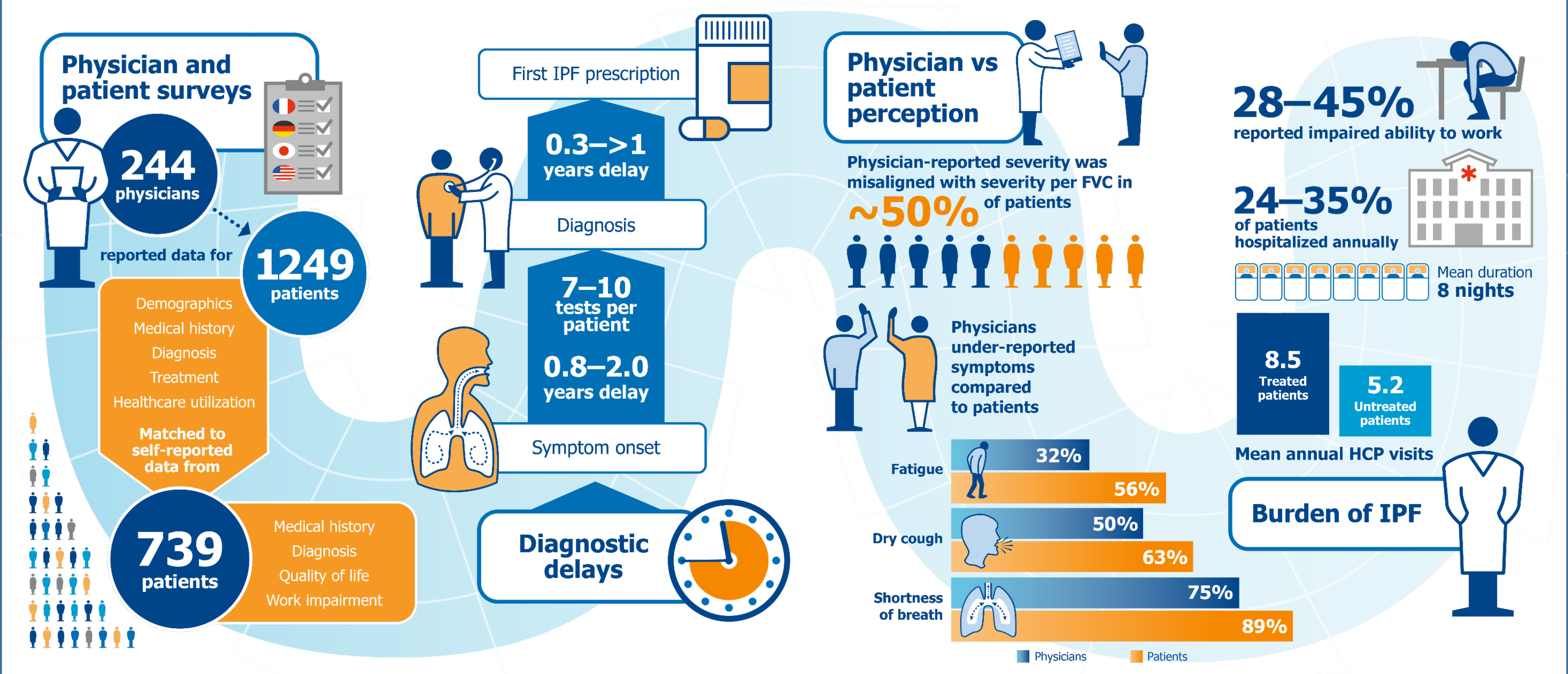

Respirology

Idiopathic pulmonary fibrosis: Physician and patient perspectives on the pathway to care from symptom recognition to diagnosis and disease burden

Lancaster et al. Respirology 2021; DOI: 10.1111/resp.14154
